# Risk factors for cooking-related burn injuries in children, WHO Global Burn Registry

**DOI:** 10.2471/BLT.20.279786

**Published:** 2021-04-01

**Authors:** Joseph S Puthumana, Ledibabari M Ngaage, Mimi R Borrelli, Erin M Rada, Julie Caffrey, Yvonne Rasko

**Affiliations:** a Department of Surgery, University of Maryland School of Medicine, 22 S Greene St, Baltimore, MD 21230, United States of America (USA).; b Division of Plastic Surgery, Stanford University, Palo Alto, USA.; c Department of Plastic & Reconstructive Surgery, Johns Hopkins University School of Medicine, Baltimore, USA.

## Abstract

**Objective:**

To assess the characteristics of cooking-related burn injuries in children reported to the World Health Organization Global Burn Registry.

**Methods:**

On 1 February 2021, we downloaded data from the Global Burn Registry on demographic and clinical characteristics of patients younger than 19 years. We performed multivariate regressions to identify risk factors predictive of mortality and total body surface area affected by burns.

**Findings:**

Of the 2957 paediatric patients with burn injuries, 974 involved cooking (32.9%). More burns occurred in boys (532 patients; 54.6%) than in girls, and in children 2 years and younger (489 patients; 50.2%). Accidental contact and liquefied petroleum caused most burn injuries (729 patients; 74.8% and 293 patients; 30.1%, respectively). Burn contact by explosions (odds ratio, OR: 2.8; 95% confidence interval, CI: 1.4–5.7) or fires in the cooking area (OR: 3.0; 95% CI: 1.3–6.8), as well as the cooking fuels wood (OR: 2.2; 95 CI%: 1.3–3.4), kerosene (OR: 1.9; 95% CI: 1.0–3.6) or natural gas (OR: 1.5; 95% CI: 1.0–2.2) were associated with larger body surface area affected. Mortality was associated with explosions (OR: 7.5; 95% CI: 2.2–25.9) and fires in the cooking area (OR: 6.9; 95% CI: 1.9–25.7), charcoal (OR: 4.6; 95% CI: 2.0–10.5), kerosene (OR: 3.9; 95% CI: 1.4–10.8), natural gas (OR: 3.0; 95% CI: 1.5–6.1) or wood (OR: 2.8; 95% CI: 1.1–7.1).

**Conclusion:**

Preventive interventions directed against explosions, fires in cooking areas and hazardous cooking fuels should be implemented to reduce morbidity and mortality from cooking-related burn injuries.

## Résumé

**Objectif:**

Définir les caractéristiques des brûlures liées à la cuisine chez les enfants, et signalées au Registre des brûlures de l'Organisation mondiale de la Santé.

**Méthodes:**

Le 1er février 2021, nous avons téléchargé les données du Registre mondial des brûlures concernant les caractéristiques démographiques et cliniques des patients âgés de moins de 19 ans. Nous avons employé un modèle de régression multivariée afin d'identifier les facteurs de risque permettant de prédire la mortalité et la surface corporelle totale touchée par les brûlures.

**Résultats:**

Sur les 2957 patients pédiatriques présentant des brûlures, 974 avaient subi des accidents domestiques liés à la cuisine (32,9%). Cela concernait plus de garçons (532 patients; 54,6%) que de filles et des enfants de 2 ans ou moins (489 patients; 50,2%). Le contact accidentel et le pétrole liquide sont à l'origine de la majorité des brûlures (729 patients; 74,8% et 293 patients; 30,1%, respectivement). Les brûlures de contact dues à des explosions (odds ratio, OR: 2.8 ; intervalle de confiance de 95%, IC: 1,4–5,7) ou les incendies dans l'espace dédié à la cuisine (OR: 3,0; IC de 95%: 1,3–6,8), ainsi que les combustibles de cuisson tels que le bois (OR: 2,2; IC de 95%: 1,3–3,4), le kérosène (OR: 1,9; IC de 95%: 1,0–3,6) ou le gaz naturel (OR: 1,5; IC de 95%: 1,0–2,2) allaient de pair avec de plus vastes surfaces corporelles affectées. La mortalité était causée par des explosions (OR: 7,5; IC de 95%: 2,2–25,9) et des incendies dans l'espace dédié à la cuisine (OR: 6,9; IC de 95%: 1,9–25,7), ainsi que des combustibles comme le charbon (OR: 4,6; IC de 95%: 2,0-10,5), le kérosène (OR: 3,9; IC de 95%: 1,4–10,8), le gaz naturel (OR: 3,0; IC de 95%: 1,5–6,1) ou le bois (OR: 2,8; IC de 95%: 1,1-7,1).

**Conclusion:**

Des actions préventives pour éviter les explosions, les incendies dans les espaces dédiés à la cuisine et les combustibles de cuisson dangereux devraient être menées afin de diminuer la morbidité et la mortalité qu'engendrent les brûlures liées à la cuisine.

## Resumen

**Objetivo:**

Evaluar las características de las lesiones por quemaduras relacionadas con la cocina en niños notificadas al Registro Mundial de Quemaduras de la Organización Mundial de la Salud.

**Métodos:**

El 1 de febrero de 2021, descargamos los datos del Registro Mundial de Quemaduras sobre las características demográficas y clínicas de los pacientes menores de 19 años. Realizamos regresiones multivariantes para identificar los factores de riesgo que predicen la mortalidad y la superficie corporal total afectada por las quemaduras.

**Resultados:**

De los 2.957 pacientes pediátricos con lesiones por quemaduras, 974 tenían que cocinar (32,9%). Se produjeron más quemaduras en niños (532 pacientes; 54,6%) que en niñas, y en niños de 2 años o menos (489 pacientes; 50,2%). El contacto accidental y el petróleo licuado causaron la mayoría de las lesiones por quemaduras (729 pacientes; 74,8% y 293 pacientes; 30,1%, respectivamente). El contacto con quemaduras por explosiones (odds ratio, OR: 2,8; intervalo de confianza del 95%, IC: 1,4-5,7) o los incendios en la zona de cocción (OR: 3,0; IC del 95%: 1,3-6,8) así como los combustibles para cocinar de madera (OR: 2,2; IC del 95%: 1,3-3,4), queroseno (OR: 1,9; IC del 95%: 1,0-3,6) o gas natural (OR: 1,5; IC del 95%: 1,0-2,2) se asociaron con una mayor superficie corporal afectada. La mortalidad se asoció a las explosiones (OR: 7,5; IC 95%: 2,2-25,9) y a los incendios en la zona de cocción (OR: 6,9; IC 95%: 1,9-25,7), el carbón vegetal (OR: 4 6; IC 95%: 2,0-10,5), queroseno (OR: 3,9; IC 95%: 1,4-10,8), gas natural (OR: 3,0; IC 95%: 1,5-6,1) o la madera (OR: 2,8; IC 95%: 1,1-7,1).

**Conclusión:**

Deben llevarse a cabo intervenciones preventivas contra las explosiones, los incendios en las zonas de cocción y los combustibles peligrosos para cocinar, a fin de reducir la morbilidad y la mortalidad por quemaduras relacionadas con la cocina.

## ملخص

الغرض تقييم خصائص إصابات الحروق المتعلقة بالطهي لدى الأطفال التي تم الإبلاغ عنها لسجل الحروق العالمي لمنظمة الصحة العالمية.

الطريقة في 1 فبراير/شباط 2021، قمنا بتنزيل بيانات من سجل الحروق العالمي حول الخصائص السكانية والإكلينيكية للمرضى الذين تقل أعمارهم عن 19 عامًا. لقد أجرينا تحوفات متعددة المتغيرات لتحديد عوامل الخطر التي تنبئ بالوفيات وإجمالي مساحة سطح الجسم المتأثرة بالحروق.

النتائج من بين 2957 إصابة لحروق المرضى من الأطفال، كانت من بينها 974 إصابة نتيجة الطبخ (32.9٪). حدث المزيد من الحروق للأولاد عن البنات (532 مريضًا؛ 54.6٪)، وللأطفالبسنسنتين أو اقل (489 مريضًا؛ 50.2٪). تسبب التلامس العرضي والنفط المسال في معظم إصابات الحروق (729 مريضاً، 74.8٪، و293 مريضاً، 30.1٪ على الترتيب). ملامسة الاحتراق عن طريق الانفجارات (نسبة الاحتمالات، 2.8؛ فاصل الثقة 95٪، 1.4 إلى 5.7)، أو الحرائق في مكان الطهي (نسبة الاحتمالات: 3؛ 95٪، 1.3 إلى 6.8)، فضلاً عن خشب وقود الطهي (نسبة الاحتمالات: 2.2؛ 95٪: 1.3 إلى 3.4)، أو الكيروسين (نسبة الاحتمالات: 1.9؛ 95٪، فاصل الثقة: 1.0 إلى 3.6)، أو الغاز الطبيعي (نسبة الاحتمالات: 1.5؛ 95٪، فاصل الثقة: 1.0 إلى 2.2) مرتبطة بمساحة أكبر لسطح الجسم المتضرر. ارتبط معدل الوفيات بالانفجارات (نسبة الاحتمالات: 7.5؛ ‏بفاصل ثقة 95%: 2.2 إلى 25.9) الحرائق في مكان الطهي (نسبة الاحتمالات: 6.9؛ ‏بفاصل ثقة 95%: 1.9 إلى 25.7)، والفحم (نسبة الاحتمالات: 4.6؛ ‏بفاصل ثقة 95%: 2.0 إلى 10.5)، والكيروسين (نسبة الاحتمالات: 3.9؛ ‏بفاصل ثقة 95%: 1.4 إلى 10.8) أو الغاز الطبيعي (نسبة الاحتمالات: 3.0؛ ‏بفاصل ثقة 95%: 1.5 إلى 6.1)، أو الخشب (نسبة الاحتمالات: 2.8؛ ‏بفاصل ثقة 95%: 1.1 إلى 7.1).

الاستنتاج يجب تنفيذ التدخلات الوقائية الموجهة ضد الانفجارات، والحرائق في أماكن الطهي، وأنواع وقود الطهي الخطيرة، وذلك للحد من معدلات الإصابة بالمرض والوفيات نتيجة إصابات الحروق المرتبطة بالطهي.

## 摘要

**目的:**

旨在评估向世卫组织全球烧伤注册平台报告的烹饪相关儿童烧伤事件的特征。

**方法:**

2021 年 2 月 1 日，我们从全球烧伤注册平台上下载了 19 岁以下患者的人口统计学和临床特征相关数据。通过进行多元回归分析，我们确定了风险因素，预测了因烧伤而导致的死亡率和全身表面积感染率。

**结果:**

在 2957 例烧伤的儿科患者中，974 例的受伤原因与烹饪有关 (32.9%)。烧伤患者中，男孩（532 例；占 54.6％）数量多于女孩数量且 2 岁及以下儿童（489 例；占 50.2％）占比较大。大多数人是因意外接触和液化石油气（分别为 729 例；占 74.8％ 和 293 例；占 30.1％）而导致被烧伤。因烹饪区爆炸（优势比，OR：2.8；95％ 置信区间，CI：1.4–5.7）或着火 (OR:3.0; 95% CI:1.3–6.8,) 以及烹饪用燃材 (OR: 2.2; 95 CI%: 1.3-3.4)、煤油 (OR: 1.9; 95% CI: 1.0-3.6） 或天然气 (OR: 1.5; 95% CI: 1.0–2.2) 导致烧伤后通常会出现全身大面积感染。因烹饪区爆炸 (OR: 7.5; 95% CI: 2.2–25.9) 和着火 (OR: 6.9; 95% CI: 1.9-25.7）、木炭 (OR: 4.6; 95% CI: 2.0-10.5）、煤油 (OR: 3.9; 95% CI: 1.4–10.8)、天然气 (OR: 3.0; 95% CI: 1.5–6.1) 或木材 (OR: 2.8; 95% CI: 1.1–7.1) 导致烧伤后通常会致死。

**结论:**

应采取可有效防止烹饪区和危险烹饪燃料发生爆炸、着火意外的预防措施，以降低因烹饪相关烧伤而导致的发病率和死亡率。

## Резюме

**Цель:**

Оценить характеристики ожоговых травм у детей, связанных с приготовлением пищи, зарегистрированные в Глобальном реестре ожогов Всемирной организации здравоохранения.

**Методы:**

1 февраля 2021 года авторы загрузили данные о демографических и клинических характеристиках пациентов младше 19 лет из Глобального реестра ожогов. Для выявления факторов риска, позволяющих прогнозировать уровень смертности и общую площадь поверхности тела, пораженную ожогами, использовали многомерный регрессионный анализ.

**Результаты:**

Из 2957 детей с ожоговыми травмами 974 занимались приготовлением пищи (32,9%). Больше ожогов было у мальчиков (532 пациента; 54,6%), чем у девочек, и детей до 2 лет (489 пациентов; 50,2%). Причиной большинства ожогов служили случайный контакт с огнем и сжиженный газ (729 пациентов; 74,8% и 293 пациента; 30,1% соответственно). Контактные ожоги в результате взрыва (показатель шансов, ПШ: 2,8; 95%-й ДИ: 1,4–5,7) или пожара в зоне приготовления пищи (ПШ: 3,0; 95%-й ДИ: 1,3–6,8), а также использования дров для приготовления пищи (ПШ: 2,2; 95%-й ДИ: 1,3–3,4), керосина (ПШ: 1,9; 95%-й ДИ: 1,0–3,6) или природного газа (ПШ: 1,5; 95%-й ДИ: 1,0–2,2) приводили к большей площади ожога. Смертность была связана со взрывами (ПШ: 7,5; 95%-й ДИ: 2,2–25,9), пожарами в зоне приготовления пищи (ПШ: 6,9; 95%-й ДИ: 1,9–25,7), использованием древесного угля (ПШ: 4,6; 95%-й ДИ: 2,0–10,5), керосина (ПШ: 3,9; 95%-й ДИ: 1,4–10,8), природного газа (ПШ: 3,0; 95%-й ДИ: 1,5–6,1) или дров (ПШ: 2,8; 95%-й ДИ: 1,1–7,1).

**Вывод:**

Для снижения уровня травматизма и смертности от ожогов, связанных с приготовлением пищи, следует осуществлять профилактические мероприятия, направленные на устранение взрывов, пожаров в зонах приготовления пищи и пожаров вследствие использования опасных видов топлива.

## Introduction

Globally, burn injuries remain among the most prominent and preventable injuries in children. Childhood burns can have devastating physical, psychological and socioeconomic consequences.^1^ Cooking-related burns have emerged as a particularly damaging subset of childhood burns, representing up to an estimated 85% of childhood scalds, and these types of burns carry a greater patient injury burden and long-term morbidity than burns of other etiologies.^2^^–^^4^ Young children and girls are more likely to experience paediatric burns.^1^^,^^5^^–^^8^ Low socioeconomic status and its associated circumstances, such as lack of water supply and volatile cooking materials, are also reported to be key risk factors for childhood cooking burns.^9^^,^^10^

Research on paediatric cooking burns has been largely restricted to local or national studies, limiting generalizability of conclusions, especially to global systemic factors underlying disparities in burn injuries.^4^^,^^5^^,^^11^^,^^12^ Wide variability in research methods has made meaningful comparisons of clinical outcomes and risk factors between high- and low-income countries difficult. While rapid declines in mortality from childhood burns have been reported in high-income countries over the past decades,^13^ the same trend has not been documented in low- and middle-income countries.^9^

We aim to address this gap in the literature by analysing data from the first large-scale, global data repository for burn injuries, the World Health Organization (WHO) Global Burn Registry.^14^ The registry, launched in January 2018, is a global instrument to standardize the reporting of hospital admissions due to burn injury across the world and was developed for public health officials and researchers after a thorough evaluation of existing national and international burn registries (Box 1). The registry comprises data submitted voluntarily by hospital staff members. A study evaluating the instrument reported that mostly physicians entered data into the system, followed by nurses.^15^ The registry records hospital admissions, injury characteristics and patient demographics. The aim of our research was to identify the key risk factors predictive of paediatric cooking burns and mortality following these burns by using the data submitted to the registry, and to inform strategies for prevention and management.

Box 1 **Description of the Global Burn Registry**

The Global Burn Registry is a global instrument developed by WHO to standardize the reporting and registry of burn admissions across the world. The registry comprises voluntary, anonymized burn data submitted by hospitals for use by public health officials and academic researchers.

Data available include: country and city of patient, WHO region, World Bank Income category, respondent (e.g. patient, sibling), age, sex, pre-hospital duration hours, burn surface area, smoke injuries, associated anatomic injuries (e.g. head and neck, trunk), cause of burn, cooking-related factors (e.g. contact method, fuel, cooking height), contributing factors, number of people injured, surgery during admission, hospital stay, condition of discharge (e.g. dead, discharged without physical impairment), submission date and health facility information.

WHO: World Health Organization.

## Methods

### Data source

We searched the Global Burn Registry for all cooking-related burns injuries on 1 February 2021. From the registry, we retrieved details about patients’ demographics, burn characteristics (e.g. total body surface area), mode of contact causing the burn injury, cooking fuel sources, hospital characteristics (e.g. length of stay) and discharge outcomes of the index admission. We de-identified and uploaded the data onto a preformatted Excel spreadsheet, Version 16.33 (Microsoft, Redmond, United States of America, USA). The data in the registry are anonymized and available to academic institutions, and therefore are not considered protected health information so institutional review board approval is not required. We followed the terms and conditions given by WHO for using the data and/or information in the registry.^16^

### Study cohort

The study cohort included all paediatric burn injuries recorded in the Global Burn Registry from 1 January 2018 to 1 February 2021. Following the National Institute of Child Health and Human Development, we considered patients younger than 19 years as paediatric patients.^17^

### Variables

The primary outcome was burn severity as determined by two proxy metrics: (i) total percentage of body surface area affected; and (ii) mortality. We assessed five primary risk factors: sex, age, World Bank income category of country of residence, mode of contact causing burn injury, and cooking fuel. We used the National Institute of Child Health and Human Development age group categories,^17^ a validated categorization of the biopsychosocial changes that occur from birth to adulthood.^18^ The age categories are: children 2 years and younger; 3–5 years; 6–11 years; and 12–18 years. We included sex and age as risk factors, since they are well-documented predictors in regional studies and therefore merit further investigation on an international scale.^5^^–^^8^ The global nature of the registry also allows study of the relationship between childhood burn injuries and socioeconomic factors, for which World Bank income classification is often used as a proxy measure.^19^ The World Bank classify countries into four groups based on their gross national income per capita: high income, upper-middle income, lower-middle income and low income.^20^ Information on the mode of contact causing the burn injury and cooking fuel used are entered for every patient with a cooking burn. These data provide valuable insight to inform preventive measures. One of the following modes of contact can be chosen when entering data: deliberate movement (e.g. deliberate touch to hot surface); accidental contact (e.g. fall/spill); exposure to open fire in the cooking area; explosion in the cooking area; or an unspecified other. Cooking fuel was categorized as: natural gas, liquefied petroleum, kerosene (paraffin), electricity, wood, charcoal and other. We categorized fuel sources that were infrequently reported in the registry as other (e.g. ethanol, traditional biomass, and unspecified sources).

### Statistical analysis

For data analysis we used SPSS software, Version 26 (IBM Corp., Armonk, USA). Frequencies and percentages were calculated for categorical variables. Medians and interquartile ranges (IQR) were calculated for continuous data. We found that total percentage of body surface area affected was a skewed continuous variable (skewness: 2.1), therefore the Kruskal–Wallis test was used to find factors associated with increased total body surface area affected and mortality, and the Mann–Whitney *U* test was used to compare subgroups. We assessed the five selected risk factors as predictors for mortality and the severity of total body surface area affected, using logistic regression. The median total body surface area affected of the cohort (15%) was used as the threshold for logistic regression. All statistical tests were considered to be significant at two-sided *P* < 0.050.

## Results

In total, 17 countries across all WHO regions reported 6965 burn injuries to the registry (Table 1). Of these, 2957 (42.4%) documented cases involved paediatric patients. Of the paediatric burn injuries, 974 (32.9%) were explicitly cooking related (Table 1). Besides cooking burns, other sources of burn injuries in the registry included washing, house fires, lighting and intentional flame burns. The median total body surface area affected by cooking burns was 15.0% (IQR: 10.0–25.0%) and the overall mortality was 8.5% (83/974; Table 2). Average duration of hospital stay was 16.4 days among patients who survived and 9.2 days among patients who died (*P* value: < 0.001). Patients who survived were more likely to have undergone surgery during their hospital stay than patients who died (44.2%; 394/891 vs 26.5%; 22/83; *P* value: 0.002).

**Table 1:** Burn injuries reported to the WHO Global Burn Registry, by country, 2018–2021

Country, by income category^a^	No.			
	Total burns	Paediatric burns	Paediatric cooking burns	Months in registry
**High income**				
Argentina	27	6	0	31
Chile	173	6	0	26
Estonia	40	1	1	26
Russian Federation	78	78	65	15
Saudi Arabia	19	10	3	22
**Upper-middle income**				
China	1	1	0	24
Iran (Islamic Republic of)	3053	1022	121	17
Mexico	357	100	31	30
Peru	835	834	297	36
South Africa	280	89	26	32
**Lower-middle income**				
India	728	175	53	29
Lao People's Democratic Republic	6	6	3	34
Nigeria	296	156	65	27
Pakistan	596	162	59	32
**Low income**				
Ethiopia	8	4	1	13
Nepal	154	42	20	36
United Republic of Tanzania	314	265	229	24
**Total**	**6965**	**2957**	**974**	**NA**

NA: not applicable; WHO: World Health Organization.

^a^ Country income category as World Bank Classification in 2021.

**Table 2:** Characteristics of children with burn injuries included in the study on identifying risk factors for cooking burns, 2018–2021

Characteristic	Patients, no. (%) (*n* = 974)		Total body surface area affected			Mortality	
			Median % (IQR)	*P*		% (no. deaths/no. admissions)	*P*
**Sex**				0.093			0.002
Male^a^	532 (54.6)		15.0 (10.0–20.0)	–		6.0 (32/532)	–
Female	442 (45.4)		15.0 (10.0–25.0)	0.093		11.5 (51/442)	0.002
**Age, years**				0.040			0.005
0–2^a^	489 (50.2)		15.0 (5.0–28.75)	–		5.5 (27/489)	–
3–5	261 (26.8)		15.0 (10.0–25.0)	0.358		11.5 (30/261)	0.003
6–11	116 (11.9)		15.0 (10.0–25.0)	0.005		9.5 (11/116)	0.114
12–18	108 (11.1)		15.0 (10.0–20.0)	0.151		13.9 (15/108)	0.002
**Income category of country of residence^b^**				0.520			0.614
High-income^a^	69 (7.1)		10.0 (5.0–25.0)	–		4.3 (3/69)	–
Upper-middle-income	475 (48.8)		15.0 (10.0–20.0)	0.237		8.8 (42/475)	0.206
Lower-middle-income	180 (18.5)		15.0 (10.0–20.0)	0.270		9.4 (17/180)	0.186
Low-income	250 (25.7)		15.0 (10.0–25.0)	0.136		8.4 (21/250)	0.259
**Mode of contact**				< 0.001			< 0.001
Deliberate touch^a^	108 (11.1)		10.0 (5.0–20.0)	–		3.7 (4/108)	–
Accidental contact	729 (74.8)		15.0 (10.0–20.0)	0.014		6.0 (44/729)	0.331
Explosion	55 (5.6)		20.0 (10.0–45.0)	< 0.001		23.6 (13/55)	< 0.001
Other	46 (4.7)		15.0 (10.0–30.0)	0.003		26.1 (12/46)	< 0.001
Fire in cooking area	36 (3.7)		22.5 (10.0–40.0)	< 0.001		27.8 (10/36)	< 0.001
**Cooking fuel**				0.048			< 0.001
Electricity^a^	85 (8.7)		15.0 (10.0–20.0)	–		0.0 (0/85)	–
Liquefied petroleum	293 (30.1)		15.0 (10.0–20.0)	0.913		4.4 (13/293)	0.048
Wood	101 (10.4)		15.0 (10.0–27.5)	0.060		8.9 (9/101)	0.005
Unspecified	9 (0.9)		10.0 (10.0–20.0)	0.543		11.1 (1/9)	0.002
Charcoal	177 (18.2)		15.0 (10.0–20.0)	0.610		11.3 (20/177)	0.001
Natural gas	259 (26.6)		15.0 (10.0–25.0)	0.301		12.4 (32/259)	0.001
Kerosene	50 (5.1)		15.0 (10.0–35.0)	0.029		16.0 (8/50)	< 0.001

IQR: interquartile range.

^a^ Reference category used for subgroup comparison.

^b^ Country income category as World Bank Classification in 2021.

Note: Inconsistencies arise in some values due to rounding. We used Kruskal–Wallis for determining group significance and Mann–Whitney *U* for subgroup significance.

### Sex and age

Burn injuries occurred more often in boys (532 patients; 54.6%) and children 2 years and younger (489 patients; 50.2%; Table 2). Girls suffered burns with a higher mortality rate (*P* value: 0.002) compared to boys. Although children 2 years and younger experienced the highest number of burns, they suffered less deadly burns than children in other age groups (vs 3–5 years *P* value: 0.003; vs 6–11 years *P* value: 0.114; vs 12–18 years *P* value: 0.002; Table 2).

### Country income classification

Most paediatric cooking burns in the registry were reported in upper-middle-income countries (475 injuries; 48.8%). There was no significant difference in total percentage of body surface area affected by burns (*P* value: 0.520) or in mortality (*P* value: 0.614) between the different income categories (Table 2).

### Mode of contact

Most children were exposed to burns by accidental contact (729 patients; 74.8%) followed by deliberate touch (108 patients; 11.1%). Compared to the least deadly burns (those caused by deliberate touch), burns caused by explosions, fires in the cooking area and unspecified other sources caused significantly higher mortality (all *P* values < 0.001). Explosions (*P* value < 0.001), fires in the cooking area (*P* value < 0.001), other sources (*P* value: 0.003) as well as accidental contact (*P* value: 0.014) caused significantly larger body surface burns than deliberate touch (Table 2).

### Cooking fuel

The most commonly used cooking fuel in childhood burns was liquefied petroleum (293 patients; 30.1%). Burns involving kerosene caused the highest mortality (eight patients; 16.0%). Compared to the least deadly cooking fuel, electricity (no deaths), all other fuels caused burns with significantly higher mortality (kerosene *P* value < 0.001; natural gas *P* value: 0.001; charcoal *P* value: 0.001; wood *P* value: 0.005; liquefied petroleum *P* value: 0.048 and other fuels *P* value: 0.002). Kerosene was the only fuel source that caused significantly larger body surface burns than electricity (*P* value: 0.029; Table 2).

### Risk factors

Children injured by a fire in the cooking area or an explosion were more likely to have more than 15% of the total body surface area affected than children who were injured by deliberate touch (odds ratio, OR: 3.0; 95% confidence interval, CI: 1.3–6.8 and OR: 2.8; 95% CI: 1.4–5.7, respectively). Compared to liquefied petroleum, the fuel sources wood (OR: 2.2; 95 CI%: 1.3–3.4), kerosene (OR: 1.9; 95% CI: 1.0–3.6) or natural gas (OR: 1.5; 95% CI: 1.0–2.2) were more likely to cause burn injuries affecting more than 15% of the total body surface area (Table 3).

**Table 3:** Risk factors associated with total burn surface area and mortality of paediatric cooking burn patients, 2018–2021

Characteristic	OR (95% CI)	
	Total burn surface area^a^	Mortality
**Sex**		
Male	1.0	1.0
Female	1.0 (0.5–2.2)	1.7 (0.5–5.3)
**Age, years**		
0–2	1.0	1.0
3–5	1.0 (0.5–2.0)	1.2 (0.4–3.8)
6–11	2.0 (0.9–4.7)	1.4 (0.4–5.6)
12–18	1.3 (0.5–3.0)	1.2 (0.3–5.2)
**Income category of country of residence^b^**		
High income	1.0	1.0
Upper-middle income	1.2 (0.7–2.2)	1.7 (0.5–6.0)
Lower-middle income	1.0 (0.5–1.8)	1.6 (0.4–6.0)
Low income	1.5 (0.8–2.7)	1.7 (0.5–6.0)
**Mode of contact**		
Deliberate touch	1.0	1.0
Accidental contact	1.3 (0.8–2.1)	1.3 (0.4–3.7)
Explosion	2.8 (1.4–5.7)	7.5 (2.2–25.9)
Other	1.9 (0.9–4.1)	6.6 (1.9–23.0)
Fire in cooking area	3.0 (1.3–6.8)	6.9 (1.9–25.7)
**Cooking fuel**		
Electricity	1.0 (0.6–1.8)	NA^c^
Liquefied petroleum	1.0	1.0
Wood	2.2 (1.3–3.4)	2.8 (1.1–7.1)
Unspecified	0.9 (0.2–4.0)	1.5 (0.1–14.7)
Charcoal	1.4 (0.9–2.1)	4.6 (2.0–10.5)
Natural gas	1.5 (1.0–2.2)	3.0 (1.5–6.1)
Kerosene	1.9 (1.0–3.6)	3.9 (1.4–10.8)

CI: confidence interval; NA: not applicable; OR: odds ratio.

^a^ The median total body surface area affected of the cohort (15%) was used as the threshold for the logistic regression analysis.

^b^ Country income category as World Bank Classification in 2021.

^c^ No deaths recorded.

Children were more likely to have died if they had been injured by an explosion (OR: 7.5; 95% CI: 2.2–25.9), a fire in the cooking area (OR: 6.9; 95% CI: 1.9–25.7) or if an unspecified contact method was reported to the registry (OR: 6.6; 95% CI: 1.9–23.0) than children who were injured by deliberate touch. Compared to liquefied petroleum, children injured by the fuel sources charcoal (OR: 4.6; 95% CI: 2.0–10.5), kerosene (OR: 3.9; 95% CI: 1.4–10.8), natural gas (OR: 3.0; 95% CI: 1.5–6.1) or wood (OR: 2.8; 95% CI: 1.1–7.1) were more likely to die (Table 3).

## Discussion

We used WHO Global Burn Registry to understand the cause of and risk factors for cooking burns in children. Unlike previous work that used single-centre and national databases, this study compared burn causes and health outcomes on an international scale to better inform and advance global surgical initiatives.

Our findings corroborate previous research on the demographics of children injured by cooking-related burns.^5^^,^^7^^,^^9^^,^^21^ Although more than half of these burns occurred in boys, girls were nearly twice as likely to die from these burns. The female predominance in burn severity may relate to the relegation of women to cooking roles and hence girls might spend more time in places with dangerous cooking fires and hot liquids than boys.^22^^–^^24^ We found that half the burn injuries reported to the registry were in children 2 years and younger. Infants are known to be disproportionally affected by all types of burn injuries.^6^^,^^25^ Younger children are more impulsive, curious and lack self-awareness, putting them at greater risk of accidental burns exposure.^9^ To prevent burn injuries in young children, caretakers should be informed about preventive measures. For example, written and pictorial education material should be available at places frequented by such caretakers.^26^ In our study sample, we found no correlation between age and burn size or severity.

Previous regional work has shown that paediatric burns are more common and more deadly in low- and middle-income countries.^9^ Here we report that burn injuries did not differ significantly by burn size or mortality across country income group, and no income group was an independent risk factor for burn severity. This contradictory finding is likely due to the skewed distribution of countries in the Global Burn Registry. Of the 974 patient reports analysed, only 68 reports are from high-income countries, and of those 65 are from the Russian Federation. Despite the vast range of middle-income countries in the registry, comparing the burden of burn injuries in countries is difficult without more data from countries that progressed the most in burn care, particularly the United States and western European countries.

The findings of the study suggest that stakeholders should focus their preventive work on the mode of contact and cooking fuel. These two factors were both significant risk factors for burn size and mortality. Burn injuries caused by explosions or fires in the cooking area were associated with the highest mortality compared to any risk factor in this study. Although the most common fuel source was liquefied petroleum, the findings suggest that this is a relatively safe cooking fuel, as is electricity. In contrast, we found kerosene to be a significant risk factor for burn mortality along with natural gas, charcoal and wood. A cooking fuel and its propensity to cause a fire or explode are likely linked to one another, and further analyses are needed to dissect this relationship. To reduce severity and mortality from burns, prevention efforts should focus on explosions and fires rather than the most common burns in scalds, spills and accidental touches. Additionally, our results indicate that preventive initiatives that shift cooking methods away from hazardous fuels, like wood and kerosene, towards safer cooking fuels, like electricity and liquefied petroleum, could result in large gains in severe burn prevention. Currently, many countries subsidize rather than disincentivize the use of fuels such as kerosene; the current analysis adds a medical perspective to the growing momentum of international and economic research advising against these subsidies.^27^^,^^28^

This study has several limitations. First, entry of data in the Global Burn Registry is voluntary and subject to reporting bias and selective data input. Although interrater reliability is potentially problematic in multicentre databases, we mitigated this risk by studying objective and quantitative variables, such as mortality and total body surface area affected. Second, the registry is a relatively new instrument, with so far limited breadth and number of hospitals involved, as well as the period of data collection. At the time of study, only 17 countries had reported data for up to 3 years. Third, our cohort of cooking-related burns came from 14 of those countries, with an uneven case distribution, limiting generalizability to socioeconomic groups as a whole. Lastly, though this study reports on possible risk factors of paediatric cooking burns, these associations are observational and no causal links can be identified from this work.

In conclusion, cooking-related burns represent a sizeable and actionable subset of burn injuries in children; in this study a third of all paediatric burns were cooking-related. Our study reinforces existing literature and suggests that policy-makers need to implement initiatives that transition fuel sources from kerosene, charcoal, wood and natural gas towards liquefied petroleum and electricity. Such actions will reduce the risk for explosions and fire in cooking areas and, in addition to protecting children, also increase the safety of all people who reside in close proximity to the hazardous fuels. 
